# Sakuranetin as a Potential Regulator of Blood Pressure in Spontaneously Hypertensive Rats by Promoting Vasorelaxation through Calcium Channel Blockade

**DOI:** 10.3390/biomedicines12020346

**Published:** 2024-02-01

**Authors:** Sujin Shin, Junkyu Park, Ho-Young Choi, Youngmin Bu, Kyungjin Lee

**Affiliations:** 1 Department of Korean Medicine, Graduate School, Kyung Hee University, Seoul 02447, Republic of Korea; sjshin04@khu.ac.kr; 2 Department of Science in Korean Medicine, Graduate School, Kyung Hee University, Seoul 02447, Republic of Korea; ojeoksan@khu.ac.kr; 3 Department of Herbal Pharmacology, College of Korean Medicine, Kyung Hee University, Seoul 02447, Republic of Korea; hychoi@khu.ac.kr (H.-Y.C.); ymbu@khu.ac.kr (Y.B.)

**Keywords:** sakuranetin, natural compound, blood pressure, hypertension, cardiovascular disease, vasorelaxation, calcium channels, angiotensin II, spontaneously hypertensive rats, hypotensive effect

## Abstract

Natural compounds, known for diverse pharmacological properties, have attracted attention as potential sources for hypertension treatment. Previous studies have revealed the hypotensive effect and vascular relaxation of prunetin, a natural compound derived from *Prunus yedoensis*. However, the potential blood pressure-lowering and vasorelaxant effects of sakuranetin, another representative compound found in plants belonging to the genus *Prunus*, have remained unexplored. We aimed to fill this gap by investigating the hypotensive and vasorelaxant effects of sakuranetin in rats. Results indicated that sakuranetin, particularly in the sakuranetin 20 mg/kg group, led to significant reductions in systolic blood pressure (SBP) and diastolic blood pressure (DBP) by −14.53 ± 5.64% and −19.83 ± 6.56% at 4 h after administration. In the sakuranetin 50 mg/kg group, the SBP and DBP decreased by −13.27 ± 6.86% and −16.62 ± 10.01% at 2 h and by −21.61 ± 4.49% and −30.45 ± 5.21% at 4 h after administration. In addition, we identified the vasorelaxant effects of sakuranetin, attributing its mechanisms to the inhibition of calcium influx and the modulation of angiotensin II. Considering its hypotensive and vasorelaxant effects, sakuranetin could potentially serve as an antihypertensive agent. However, further research is required to evaluate the safety and long-term efficacy.

## 1. Introduction

Cardiovascular diseases (CVDs) are the leading causes of mortality worldwide, with an escalating prevalence observed in both developed and developing nations [1]. Hypertension, a prevalent CVD, is the strongest risk factor for CVDs such as stroke and myocardial infarction [2]. Hypertension is diagnosed when the systolic blood pressure (SBP) is ≥140 mmHg and the diastolic blood pressure (DBP) is ≥90 mmHg [3]. Uncontrolled high BP contributes to the development of chronic diseases, such as other CVDs and chronic kidney disease [4]. Accordingly, maintaining the BP within an optimal range is important. However, the incidence of hypertension is increasing due to certain factors such as obesity, excessive alcohol and salt intake, decreased physical activity, poor nutrition, and psychological stress [5].

Given that high BP often manifests without noticeable symptoms, the implementation of preventive measures is crucial [4]. In modern medicine, efforts to address hypertension include lifestyle modifications and the use of antihypertensive medications [6]. Despite these strategies, only approximately one-third of individuals in the United States maintain their target BP (SBP/DBP < 140/90 mmHg) [7]. Additionally, antihypertensive medications may induce side effects such as fatigue, headache, palpitations, and cough [7]. Therefore, relevant studies are being conducted to identify novel treatments for high BP, particularly from natural compounds.

Natural compounds derived from medicinal plants have emerged as promising candidates for CVD treatment owing to their diverse pharmacological properties, which include antioxidant, anti-inflammatory, lipid-lowering, antithrombotic, and immunomodulatory effects [8,9]. Notably, natural compounds have demonstrated significant BP-lowering effects, with 82 natural compounds approved as antihypertensive agents by the Food and Drug Administration from 1981 to 2019 [10,11]. Moreover, these compounds exhibit relaxing effects, primarily through nitric oxide (NO)/cGMP (cyclic 3′,5′-guanosine monophosphate) pathways or by blocking the Ca^2+^ channels in the vascular smooth muscle cells (VSMCs), showcasing the therapeutic potential for hypertension [12,13]. Given that some patients with high BP may not respond to or comply with conventional treatment medications, discovering new compounds for hypertension management is crucial [10].

Previous studies revealed the blood vessel-relaxing effect of the *Prunus yedoensis* bark [14]. Furthermore, the natural compound prunetin, derived from *Prunus yedoensis*, demonstrated vascular relaxant and BP-lowering effects in rats [15]. Alongside prunetin, sakuranetin is a representative compound in plants of the genus Prunus [16,17]. Sakuranetin, a flavonoid, is not only exclusive to the *Prunus* genus but is also present in various plant species, including the *Baccharis*, *Juglans*, and *Rhus* genera [16]. Moreover, sakuranetin can be synthesized by methylation of naringenin [18]. Recent studies have revealed sakuranetin’s various pharmacological effects, including antioxidant, anti-inflammatory, antiviral, antifungal, neuroprotective, antimelanogenic, and antitumor effects [19]. However, despite these findings, no study has reported its vascular relaxation or BP-lowering effects. Thus, we aimed to investigate whether sakuranetin is a potential drug for treating hypertension. Its acute BP-lowering effect was examined in spontaneously hypertensive rats (SHRs) by the tail cuff method. Moreover, its vascular relaxation effects were explored by observing the aortic rings of Sprague–Dawley (SD) rats.

## 2. Materials and Methods

### 2.1. Chemicals and Solution Preparation

Sakuranetin (≥98.0% purity as verified by high-performance liquid chromatography, Cas No: 2957-21-3) was purchased from Biopurify Phytochemicals (Chengdu, China). Dimethyl sulfoxide (DMSO) was purchased from Junsei (Tokyo, Japan). Sodium chloride (NaCl), potassium chloride (KCl), calcium chloride (CaCl_2_), magnesium sulfate (MgSO_4_), monobasic potassium phosphate (KH_2_PO_4_), sodium hydrogen carbonate (NaHCO_3_), glucose, barium chloride (BaCl_2_), and urethane were purchased from Daejung Chemicals & Metals Co. Ltd. (Siheung-si, Republic of Korea). Phenylephrine (PE), acetylcholine (ACh), ethyleneglycol-bis(2-aminoethylether)-N,N‚N,’N′-tetraacetic acid (EGTA), 2-aminoethyl diphenylborinate (2-APB), and angiotensin II (Ang II) were purchased from Sigma Aldrich (ST. Louis, MA, USA). 4-aminopyridine (4-AP) and tetraethylammonium (TEA) were purchased from Wako Pure Chemical Industries (Osaka, Japan).

Krebs–Henseleit (KH) solution used was composed of 118.0 mmol/L of NaCl, 4.7 mmol/L of KCl, 2.5 mmol/L of CaCl_2_, 1.2 mmol/L of MgSO_4_, 1.2 mmol/L of KH_2_PO_4_, 25.0 mmol/L of NaHCO_3_, and 11.1 mmol/L of glucose.

### 2.2. Experimental Animals

Male 24-week-old SHRs were obtained from SLC, Inc. (Shizuoka, Japan), while male SD 6–7-week-old rats were obtained from Daehan Biolink (Eumseong-gun, Republic of Korea). The animals were housed under constant conditions (temperature: 22 ± 2 °C, humidity: 45–65%, and a 12-h light/dark cycle), with *ad libitum* access to a standard commercial diet and water. All experiments were performed in accordance with the Animal Welfare Guidelines and approved by the Animal Experiment Ethics Committee of Kyung Hee University (KHSASP-23-506).

### 2.3. Measurement of BP

The SHRs were divided into four groups: the control group (DMSO, i.p. injection) and the sakuranetin groups (10 mg/kg, 20 mg/kg, and 50 mg/kg dissolved in DMSO; i.p. injection). BP was measured following the tail-cuff method as described previously [20]. The SBP and DBP were measured before the administration and at 1, 2, 4, and 8 h after the administration.

### 2.4. Measurement of Vascular Relaxation and Assessment of the Mechanisms Involved

#### 2.4.1. General Process of Isolating and Preparing Rat Thoracic Aortic Rings

The general process for isolating and preparing rat thoracic aortic rings has been described previously [20]. The thoracic aorta was rapidly dissected from anesthetized SD rats and cut into rings 2–3 mm in length. The endothelium-free rings were prepared by rubbing the endothelium of the vascular rings with a cotton swab. Each ring was suspended in a chamber filled with KH solution, and changes in the tension of each ring were measured and recorded. Following an initial equilibration period of 40–50 min, each of the experiments described below was conducted. 

#### 2.4.2. Measurement of Vascular Relaxation in Aortic Rings

To assess the vasorelaxant effect, sakuranetin (1, 2, 5, 10, and 20 μg/mL, dissolved in DMSO) was added to KH solution in each chamber after the rings reached the maximum contraction induced by PE (1 μM). Relaxation was expressed as the percentage of PE-induced constriction.

#### 2.4.3. Measurement of Vascular Relaxation Depending on the Presence or Absence of Endothelium

To verify endothelial integrity, blood vessel-relaxing response to ACh (10 μM) was assessed in each aortic ring pretreated with PE (1 μM). PE-constricted rings demonstrating ≥85% relaxation were classified as endothelium-present rings, while those demonstrating a vasorelaxant response of <10% were considered endothelium-absent rings. The rings were subsequently washed, and constriction was reinduced with PE (1 μM). The ensuing blood vessel-relaxing effect, depending on the presence or absence of endothelial cells, was compared.

#### 2.4.4. Measurement of Inhibitory Effect of Sakuranetin on Extracellular Ca^2+^-Induced Contraction

After isolating the thoracic aorta, the aortic rings were stabilized in a Ca^2+^-free KH solution containing EGTA (1 mM). After the equilibration period, the rings were incubated with or without sakuranetin (5, 20 μg/mL) for 20 min. The rings were pretreated with PE (1 µM) or KCl (60 mM) for another 20 min to activate the receptor-operative Ca^2+^ channels (ROCCs) or voltage-dependent Ca^2+^ channels (VDCCs). Vascular contraction response was induced by exposure to varying doses of extracellular CaCl_2_ (0.1, 0.3, 1, 3, or 10 mM), and the degree of vasoconstriction was compared between the sakuranetin-treated and non-treated groups.

#### 2.4.5. Measurement of the Inhibitory Effect of Sakuranetin on Intracellular Ca^2+^ Release

The rings were incubated with an inositol trisphosphate receptor (IP_3_R) blocker (2-APB, 10 µM) for 20 min before the induction of vasoconstriction by PE (1 µM). After the blood vessels reached the maximum contraction induced by PE, sakuranetin (1, 2, 5, 10, and 20 μg/mL) was added cumulatively to each chamber to determine the inhibitory effect of sakuranetin on intracellular Ca^2+^ release.

#### 2.4.6. Measurement of Vascular Relaxation in Aortic Rings Preincubated with K^+^ Channel Blockers

The rings were incubated with a voltage-dependent K^+^ channel blocker (4-AP, 1 mM), inward rectifier K^+^ channel blocker (BaCl_2_, 10 μM), or large-conductance Ca^2+^-activated K^+^ channel blocker (TEA, 1 mM) for 20 min before the induction of vasoconstriction by PE (1 µM). After the blood vessels reached the maximum contraction induced by PE, sakuranetin (1, 2, 5, 10, and 20 μg/mL) was administered at cumulative concentrations to explore the potential correlation between the vasorelaxation of sakuranetin and K^+^ channels.

#### 2.4.7. Measurement of Inhibitory Effect of Sakuranetin on Ang II

The rings were pretreated with or without sakuranetin (20 μg/mL) for 20 min. Subsequently, vascular contraction response was induced by the serial addition of increasing concentrations of Ang II (10^−9^, 10^−8^, 10^−7^, and 10^−6^ M), and the vasoconstriction between the sakuranetin-treated and non-treated groups was compared.

### 2.5. Statistical Analysis

Statistical analyses were performed using the GraphPad Prism 9 software (San Diego, CA, USA). All values in this study were expressed as the means ± standard error of the mean (SEM). The differences between groups were analyzed using multiple unpaired *t*-tests and two-way analysis of variance, followed by Dunnett’s post-hoc test. A *p* value of <0.05 was considered significant.

## 3. Results

### 3.1. Acute BP-Lowering Effect of Sakuranetin in SHR

**Figure 1:**
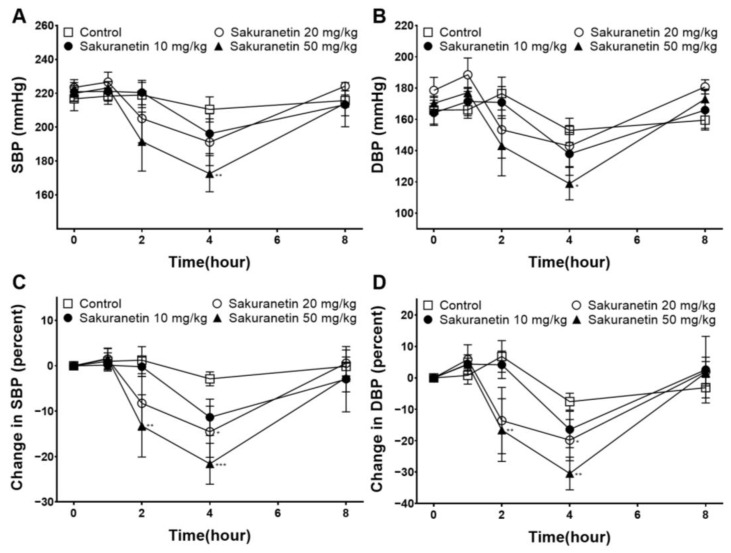
Acute blood pressure-lowering effect of sakuranetin (10, 20, and 50 mg/kg) in spontaneously hypertensive rats. (**A**) Systolic blood pressure (SBP), (**B**) diastolic blood pressure (DBP), (**C**) percent changes in SBP, and (**D**) percent changes in DBP. Values are expressed as the mean ± SEM (*n* = 5–6). * *p* < 0.05, ** *p* < 0.01, *** *p* < 0.001 vs. control.

To investigate the acute hypotensive effect of sakuranetin, BP was measured before and at 1, 2, 4, and 8 h after sakuranetin administration (10, 20, and 50 mg/kg) (Figure 1A,B). The sakuranetin 50 mg/kg group demonstrated an SBP of 172.53 ± 10.69 mmHg and a DBP of 118.86 ± 10.37 mmHg at 4 h after administration, both of which were confirmed to be lower than those observed in the control group (SBP/DBP: 210.47 ± 7.46/152.91 ± 7.77 mmHg). The difference between the BP levels measured before and after administration was calculated for each rat and expressed as a percentage (Figure 1C,D). The sakuranetin 20 mg/kg group showed a significant decrease in the percent changes of SBP (−14.53 ± 5.64%) and DBP (−19.83 ± 6.56%) at 4 h after administration. In addition, the sakuranetin 50 mg/kg group showed a significant decrease in SBP (−13.27 ± 6.86%) and DBP (−16.62 ± 10.01%) at 2 h after administration. The highest reduction in SBP (−21.61 ± 4.49%) and DBP (−30.45 ± 5.21%) was observed at 4 h after administration in the sakuranetin 50 mg/kg group.

### 3.2. Blood Vessel-Relaxing Effect of Sakuranetin on Thoracic Aortic Rings

**Figure 2:**
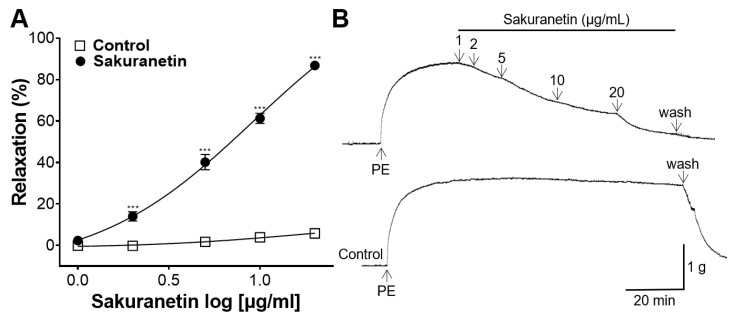
Blood vessel-relaxing effect of sakuranetin on thoracic aortic rings preconstricted with phenylephrine (PE, 1 μM). (**A**) Concentration–response curves and (**B**) representative traces. Values of relaxation (mean ± SEM) are expressed as a percentage of the constriction induced by PE (*n* = 5). *** *p* < 0.001 vs. control.

To assess the blood vessel-relaxing effect of sakuranetin, experiments examining vascular reactivity using isolated rat thoracic aortic rings were conducted. The results revealed a significant relaxing effect of sakuranetin on aortic rings preconstricted with PE compared with the control group (Figure 2). Sakuranetin exhibited a dose-dependent vasorelaxant effect, with respective values of 2.36 ± 1.07% at 1 μg/mL, 13.99 ± 2.24% at 2 μg/mL, 40.18 ± 3.69% at 5 μg/mL, 61.34 ± 2.46% at 10 μg/mL, and 86.84 ± 1.07% at 20 μg/mL.

### 3.3. Blood Vessel-Relaxing Effect of Sakuranetin on Aortic Rings in the Presence and Absence of Endothelium

**Figure 3:**
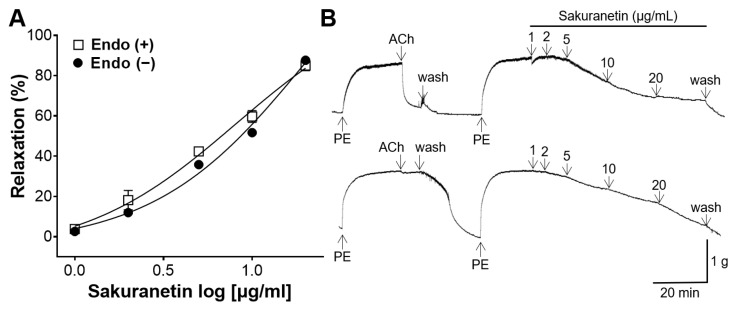
Blood vessel-relaxing effect of sakuranetin on thoracic aortic rings preconstricted with phenylephrine (PE, 1 μM) in the presence and absence of endothelium. (**A**) Concentration–response curves and (**B**) representative traces. Values of relaxation (mean ± SEM) are expressed as a percentage of the constriction induced by PE (*n* = 5).

To investigate the potential involvement of endothelial cell-related mechanisms in the blood vessel relaxation effect of sakuranetin, the vasorelaxant effect of sakuranetin was compared between two groups: endothelium-intact and endothelium-removed aortic ring groups. In the endothelium-intact aortic rings preconstricted with PE, sakuranetin exhibited a dose-dependent vasorelaxing effect, with a maximal value of 85.02 ± 2.57% at 20 μg/mL. Similarly, in aortic rings lacking endothelium, sakuranetin demonstrated a dose-dependent vasorelaxing effect, with a maximal value of 87.65 ± 0.94% at 20 μg/mL (Figure 3). Notably, no significant difference was found in the vasorelaxant effect of sakuranetin between the rings with and without endothelial cells.

### 3.4. Inhibitory Effect of Sakuranetin on Rat Aortic Rings Constricted with Extracellular Ca^2+^

**Figure 4:**
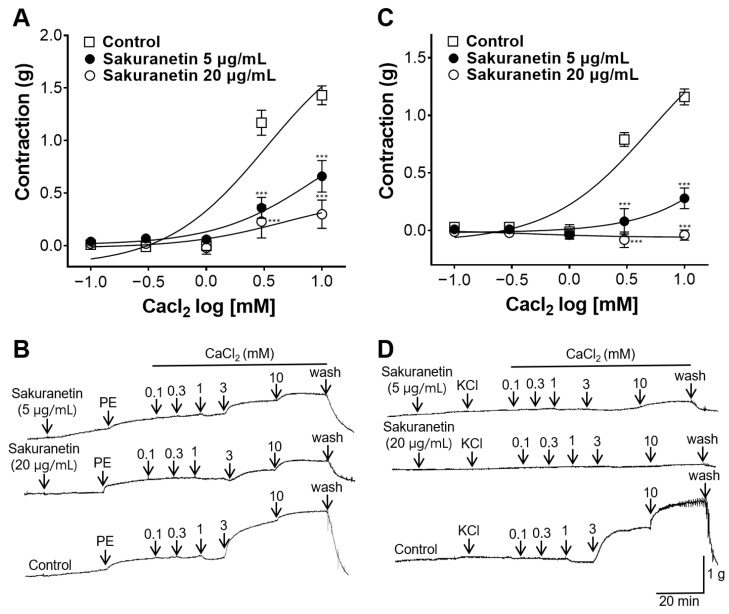
Inhibitory effect of sakuranetin on the thoracic aortic rings constricted with extracellular CaCl₂ (0.1, 0.3, 1, 3, and 10 mM). (**A**,**C**) Inhibitory effect of sakuranetin on receptor-operative Ca^2+^ channel (ROCC)- or voltage-dependent Ca^2+^ channel (VDCC)-activated rings treated with phenylephrine (PE, 1 μM) or potassium chloride (KCl, 60 mM). (**B**,**D**) Representative traces of the aortic rings. Values are expressed as the mean ± SEM (*n* = 5). *** *p* < 0.001 vs. control.

To elucidate the potential involvement of extracellular Ca^2+^ influx through Ca^2+^ channels in the blood vessel-relaxing effect of sakuranetin, vasoconstriction was induced by adding Ca^2+^ to the aortic rings. The resulting vasoconstriction was compared between the sakuranetin-treated and the untreated groups. Treatment with sakuranetin (5 and 20 μg/mL) showed reduced contraction in the ROCC-activated aortic rings compared with the untreated group (Figure 4A,B). Additionally, sakuranetin treatment (5 and 20 μg/mL) demonstrated reduced constriction in VDCC-activated aortic rings treated with KCl (Figure 4C,D).

### 3.5. Inhibitory Effect of Sakuranetin on Intracellular Ca^2+^ Release

**Figure 5:**
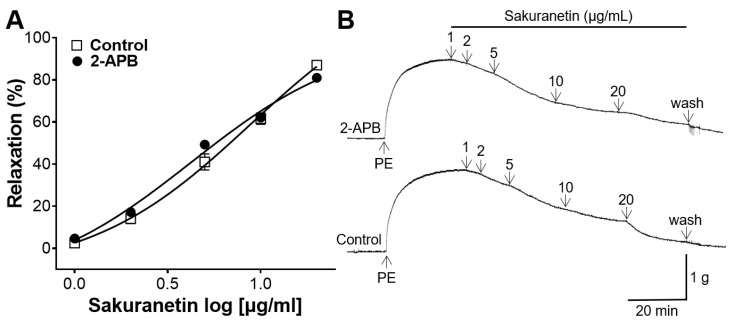
Blood vessel-relaxing effects of sakuranetin on thoracic aortic rings pretreated with 2-aminoethyl diphenylborinate (2-APB, 10 µM). (**A**) Concentration–response curves and (**B**) representative traces. Values of relaxation (mean ± SEM) are expressed as a percentage of the constriction induced by PE (*n* = 5).

To assess the impact of sakuranetin on intracellular Ca^2+^ release, pretreatment with 2-APB was performed before inducing PE contraction. The vasodilatory effect of sakuranetin in the group pretreated with 2-APB was compared with that in the untreated group. The 2-APB pretreatment group showed no significant difference when compared with the control group (Figure 5).

### 3.6. Blood Vessel-Relaxing Effect of Sakuranetin on Aortic Rings Preincubated with K^+^ Channel Blockers

**Figure 6:**
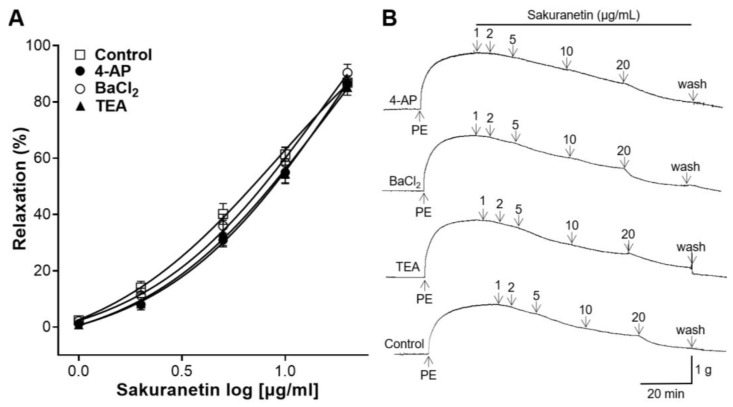
Blood vessel-relaxing effects of sakuranetin on thoracic aortic rings pretreated with 4-aminopyridine (4-AP, 1 mM), barium chloride (BaCl_2_, 10 μM), or tetraethylammonium (TEA, 1 mM). (**A**) Concentration–response curves and (**B**) representative traces. Values of relaxation (mean ± SEM) are expressed as a percentage of the constriction induced by phenylephrine (PE, 1 μM) (*n* = 5).

To investigate the potential involvement of K^+^ channels in the vasorelaxant effect of sakuranetin, pretreatment with the specific K^+^ channel blockers, including the voltage-dependent K^+^ channel blocker (4-AP), inward rectifier K^+^ channel blocker (BaCl_2_), and large-conductance Ca^2+^-activated K^+^ channel blocker (TEA), was performed before PE constriction. The vasodilatory effect of sakuranetin was compared between the group pretreated with K^+^ channel blockers and untreated group. However, the 4-AP, BaCl_2_, and TEA pretreatment groups showed no significant differences when compared with the control group (Figure 6).

### 3.7. Inhibitory Effect of Sakuranetin on Rat Aortic Rings Constricted with Ang II

**Figure 7:**
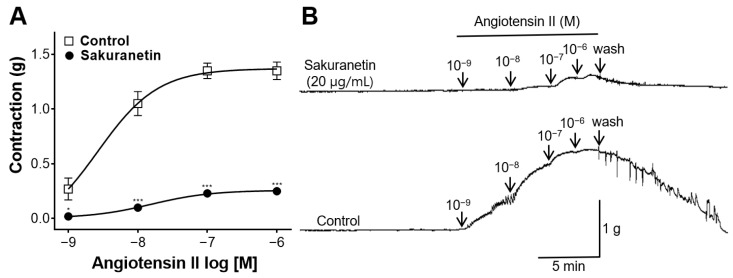
Inhibitory effect of sakuranetin on thoracic aortic rings constricted with angiotensin II (Ang II, 10^−9^−10^−6^ M). (**A**) Inhibitory effect of sakuranetin and (**B**) representative traces of aortic rings. Values are expressed as the mean ± SEM (*n* = 5). * *p* < 0.05, *** *p* < 0.001 vs. control.

To assess the inhibitory impact of sakuranetin on Ang II, vasoconstriction was induced by adding Ang II to the aortic rings. The resulting vasoconstriction was then compared between the sakuranetin-treated and untreated groups. Notably, sakuranetin (20 μg/mL) inhibited the Ang II-induced constriction of the aortic rings (Figure 7).

## 4. Discussion

In this study, we investigated the potential utility of sakuranetin in the treatment of hypertension, a prevalent CVD and a major risk factor for CVDs. Our initial investigation focused on evaluating whether sakuranetin could effectively lower the BP in SHRs. The results revealed a significant decrease in both SBP (14.53 ± 5.64%) and DBP (19.83 ± 6.56%) in the sakuranetin 20 mg/kg group at 4 h after administration. In the sakuranetin 50 mg/kg group, the SBP and DBP decreased significantly by −13.27 ± 6.86% and −16.62 ± 10.01% at 2 h and by −21.61 ± 4.49% and −30.45 ± 5.21% at 4 h after administration, respectively. The BP-lowering effect was more pronounced in the high-dose sakuranetin group, and no side effects were observed in any of the SHR groups. Compounds demonstrating a hypotensive effect in SHRs are well-documented for their ability to reduce BP levels in hypertensive individuals [21]. Therefore, our experimental outcomes suggest the potential utility of sakuranetin in treating hypertension and reducing the risk of CVDs. However, given the short-term nature of our experiment, a comprehensive understanding of sakuranetin’s effects and toxicity necessitates further experimental data. Long-term studies, encompassing diverse concentrations, are imperative to elucidate the sustained impact of sakuranetin and ensure a more comprehensive evaluation of its therapeutic potential in hypertension management.

The SHRs serve as a model for essential hypertension, representing the predominant form of high BP. Typically, the extent of BP reduction is more pronounced in SHRs when the antihypertensive therapy is administered at an early age and continued over an extended period [22]. In our experimental results, the BP-lowering effect was measured with SHRs with established hypertension, with an SBP of 210–230 mmHg. The administration of sakuranetin at doses of 20 mg/kg and 50 mg/kg resulted in SBP reductions of −14.53 ± 5.64% and −21.61 ± 4.49%, respectively, in SHRs. Consistent adherence to antihypertensive therapy has been shown to enhance the BP control [23]. Consequently, the prolonged consumption of sakuranetin may yield greater effectiveness over time.

Impaired blood vessel function, leading to inadequate dilation, may contribute to the development of hypertension [24]. Typically, vascular relaxation experiments involve the use of rat thoracic aorta, with SD or Wistar rats being the primary subjects [25]. Therefore, this study aimed to investigate whether sakuranetin possesses a blood vessel-relaxing effect on the aortic rings constricted with PE in SD rats. In our experimental results, sakuranetin (1, 2, 5, 10, and 20 μg/mL) demonstrated a dose-dependent vasorelaxant effect. Endothelial cells play an important role in modulating vascular tone by releasing mediators such as NO or prostacyclin (PGI_2_) [26]. NO and PGI_2_ mediate vasodilation through the activation of soluble guanylyl cyclase and adenyl cyclase in VSMCs, producing the cGMP and adenosine monophosphate, respectively [27]. Therefore, we investigated whether the blood vessel-relaxing effect of sakuranetin is endothelium-dependent. Vasorelaxant effects were observed in both endothelium-intact and endothelium-denuded aortic rings, with no significant difference between the two groups. Therefore, our experimental results showed that the blood vessel-relaxing effect of sakuranetin was not associated with endothelium-related mechanisms.

Intracellular Ca^2+^ also plays a pivotal role in the modulation of vascular tone, with changes in Ca^2+^ mediated by facilitating influx via the transmembrane Ca^2+^ channels (ROCCs and VDCCs) and Ca^2+^ release from intracellular stores [28]. To explore whether sakuranetin is involved in the blockade of ROCCs or VDCCs, aortic rings were preincubated with PE or KCl, and CaCl_2_ was added cumulatively to induce constriction. PE induces vasoconstriction by facilitating Ca^2+^ influx through the ROCCs and the release of Ca^2+^ from the sarcoplasmic reticulum. KCl-induced vasoconstriction is primarily attributed to the influx of Ca^2+^ upon depolarization of the cell membrane, activating VDCCs [29]. The sakuranetin treatment group (5 and 20 μg/mL) showed reduced contraction in the ROCC- and VDCC-activated aortic rings compared with the non-treated group. Therefore, sakuranetin inhibited Ca^2+^ influx by blocking ROCCs and VDCCs.

The release of Ca^2+^ from the sarcoplasmic reticulum via the IP_3_R and ryanodine receptors results in increased intracellular Ca^2+^, contributing to VSMC contraction [28]. To investigate whether sakuranetin treatment modified the intracellular Ca^2+^ release, the rings were preincubated with the IP_3_R blocker, 2-APB. However, the 2-APB pretreatment group showed no significant difference in the vasorelaxant effect compared with the control. Our data revealed that sakuranetin had no significant effect on intracellular Ca^2+^ release.

Mechanistically, the activation of K^+^ channels on the membranes of VSMCs plays a crucial role in determining membrane potential and the concentration of cytosolic Ca^2+^, resulting in vasorelaxation [30]. Various types of K^+^ channels are expressed in VSMCs. To investigate whether the blood vessel-relaxing effect of sakuranetin is related to the opening of K^+^ channels, thoracic aortic rings were preincubated with a voltage-dependent K^+^ channel blocker (4-AP), inward rectifier K^+^ channel blocker (BaCl_2_), or large-conductance Ca^2+^-activated K^+^ channel blocker (TEA). The 4-AP, BaCl_2_, and TEA pretreatment groups showed no significant differences in the vasorelaxant effect compared with the control. Therefore, our study suggests that vasorelaxation induced by sakuranetin is not associated with K^+^ channels.

Ang II, an important member of the renin–angiotensin system family, regulates blood vessel tone and volume and is a potent vasoconstrictor that elevates BP [31]. Current antihypertensive medications regulate BP levels and vascular tone using Ang II receptor blockers that impede receptor binding or angiotensin-converting enzyme inhibitors that hinder Ang II production [6]. The current study explored whether sakuranetin could mitigate the vasoconstrictive effect of Ang II, and the results revealed a significant difference in the degree of blood vessel constriction between those pretreated with sakuranetin and the control. Consequently, our findings suggest that sakuranetin inhibits Ang II activity. However, future studies are required to investigate the mechanism by which sakuranetin interferes with the action of Ang II.

Initiating antihypertensive therapy as early as possible and sustaining it is crucial in hypertensive patients, as a reduction in SBP contributes to preventing cardiac complications associated with hypertension [6,32]. Natural compounds harbor substantial potential for providing overwhelming protection against degenerative diseases, including CVDs, owing to their diverse mechanisms of action and targets [9,33]. In particular, natural compounds mainly exhibit vascular relaxation effects through various mechanisms such as the NO/cGMP pathway, the eicosanoid system, the opening of K^+^ channels, and the blockage of Ca^2+^ channels [25]. Our previous study revealed the vasorelaxant effect of prunetin by blocking Ca^2+^ channels, and our result showed that sakuranetin has the same vascular relaxation mechanism [15]. In addition, sakuranetin exhibits a vascular relaxation effect by inhibiting the activity of Ang II. Given that the effective management of high BP often involves prescribing antihypertensive drugs with various mechanisms [34], the singular nature of sakuranetin, coupled with its multiple mechanisms, positions it as a promising candidate for treating and preventing high BP. However, further studies are warranted to investigate the interaction between sakuranetin and other common antihypertensive medications.

## 5. Conclusions

Consequently, sakuranetin treatment led to a significant decrease in the SBP and DBP levels of SHRs, confirming its hypotensive effect in the essential hypertension model. In addition, we identified the vasodilatory effect of sakuranetin, which involved the inhibition of Ca^2+^ influx and the action of Ang II. Considering its BP-lowering and blood vessel-relaxing effects, sakuranetin could potentially serve as an antihypertensive agent. However, more detailed research is required to evaluate the safety, long-term efficacy, and adverse effects associated with its use.

## Data Availability

The data presented in this study are available from the corresponding author upon request.
